# Dietary Supplementation with Rutin Alters Meat Quality, Fatty Acid Profile, Antioxidant Capacity, and Expression Levels of Genes Associated with Lipid Metabolism in Breast Muscle of Qingyuan Partridge Chickens

**DOI:** 10.3390/foods12122302

**Published:** 2023-06-07

**Authors:** Yuanfei Li, Huadi Mei, Yanchen Liu, Zhenming Li, Hammad Qamar, Miao Yu, Xianyong Ma

**Affiliations:** 1 Institute of Animal Science, Guangdong Academy of Agricultural Sciences, State Key Laboratory of Livestock and Poultry Breeding, Key Laboratory of Animal Nutrition and Feed Science in South China, Ministry of Agriculture and Rural Affairs, Guangdong Provincial Key Laboratory of Animal Breeding and Nutrition, Guangdong Engineering Technology Research Center of Animal Meat Quality and Safety Control and Evaluation, Guangzhou 510640, China; li-yuan-fei@outlook.com (Y.L.); m15973699341@163.com (H.M.); mryanchenliu@outlook.com (Y.L.); lzm900120@163.com (Z.L.); drhammadqamar@gmail.com (H.Q.); yumiao@gdaas.cn (M.Y.); 2 Maoming Branch, Guangdong Laboratory for Lingnan Modern Agricultural, Maoming 525000, China

**Keywords:** rutin supplementation, antioxidant capacity, fatty acid profile, lipid metabolism, meat quality, Chinese yellow-feathered broiler, Qingyuan partridge chickens

## Abstract

Consumer demand for tasty and quality meat has been quickly increasing. This study investigated how dietary supplemented rutin affects meat quality, muscle fatty acid profile, and antioxidant capacity in the Chinese indigenous Qingyuan partridge chicken. A cohort of 180 healthy 119-day-old chickens was subjected to a randomized assignment into three groups, identified as the control, R200, and R400 groups, with respective supplementation of 0, 200, and 400 mg/kg of rutin. The results revealed insignificance in growth performance, namely, average daily gain, average daily feed intake, and feed-to-gain ratio, across the various treatment groups (*p* > 0.05). Nevertheless, dietary rutin supplementation increased (*p* < 0.05) breast muscle yield and intramuscular fat content in breast muscle and decreased (*p* < 0.05) drip loss in breast muscle. Rutin supplementation increased (*p* < 0.05) the content of high-density lipoprotein but decreased (*p* < 0.05) the contents of glucose, triglyceride, and total cholesterol in serum. Rutin supplementation increased (*p* < 0.05) the levels of DHA (C22:6*n*-3), total polyunsaturated fatty acids (PUFAs), *n*-3 PUFAs, decanoic acid (C10:0), the activity of Δ5 + Δ6 (22:6 (*n* − 3)/18:3 (*n* − 3)), and the ratio of PUFA/SFA in breast muscle but decreased (*p* < 0.05) the level of palmitoleic acid (C16:1n-7), the ratio of *n*-6/*n*-3 PUFAs, and the activity of Δ9 (16:1 (*n* − 7)/16:0). Rutin treatment also reduced (*p* < 0.05) the contents of malondialdehyde in serum and breast muscle, and increased (*p* < 0.05) the catalase activity and total antioxidant capacity in serum and breast muscle and the activity of total superoxide dismutase in serum. Additionally, rutin supplementation downregulated the expression of *AMPKα* and upregulated the expression of *PPARG*, *FADS1*, *FAS*, *ELOVL7*, *NRF2,* and *CAT* in breast muscle (*p* < 0.05). Convincingly, the results revealed that rutin supplementation improved meat quality, fatty acid profiles, especially *n*-3 PUFAs, and the antioxidant capacity of Qingyuan partridge chickens.

## 1. Introduction

Poultry meat is among the most common animal sources of food worldwide and is a key and high-quality source of protein for humans. Recently, owing to the superior meat quality of yellow-feathered broilers in comparison to white-feathered broilers, as well as the consumer preference for both wholesomeness and a pleasant flavor profile, the production of yellow-feathered broilers has been experiencing a steady upward trend in China, the second-largest producer of chicken meat in the world, and has now reached a level of output that is comparable to that of white-feathered broilers [1,2]. The Qingyuan partridge chicken, a well-known slow-growing Chinese indigenous yellow-feathered broiler, is famous for its superior meat quality and is highly popular in China and in neighboring countries [3]. However, production costs are relatively high due to the slow growth performance. Additionally, Ju et al. [4] found that the contents of polyunsaturated fatty acids (PUFAs) and DHA (C22:6n-3) in the breast muscle of Qingyuan partridge chicken were significantly lower than those in Anka chicken, recessive white feather broiler, Wenchang chicken, and Beijing fatty chicken. The PUFAs, especially *n*-3 PUFAs, are considered functional components linked to the prevention of cardiovascular disease, type 2 diabetes, and other diseases [5], resulting in increasing consumers’ preference for meat with a relatively high content of *n*-3 PUFAs. Hence, enhancing the growth performance of Qingyuan partridge chicken and optimizing the fatty acid (FA) profile of meat will further augment both production levels and consumers’ preference for the product. However, very few studies have focused on improving the growth performance and the FA profile in the muscle of Qingyuan partridge chickens.

Recently, phytopolyphenols have been utilized as natural and safe feed additives capable of enhancing growth and meat quality in livestock production. Emerging evidence shows that flavonoids, a subclass of polyphenols, can improve not only the growth performance [6] but also the deposition of *n*-3 PUFAs in chickens [7]. Rutin (3, 3′, 4′, 5, 7-pentahydrohyflavone-3-rhamnoglucoside), one of the cheapest flavonoids and abundantly distributed in food plants, drew much attention due to its tremendous health benefits and relatively low price in animal nutrition. Studies showed that rutin could facilitate the growth performance in broilers [8,9]. Additionally, rutin has been shown to have valid anti-diabetic activity and improve the anti-oxidative capacity and plasma lipid profiles in type 2 diabetic rats [10]. Dietary rutin supplementation was found to decrease the serum total cholesterol (TC) and triglyceride (TG) contents in broilers [11] and increase the serum anti-oxidative capacity in broilers [8]. These studies suggested that rutin can enhance the antioxidant capacity and improve the lipid and FA profile in serum under both normal and pathological conditions. However, whether dietary rutin supplementation affects the growth performance, antioxidant capacity, FA profile, and, thus, the quality of Qingyuan partridge chicken meat remains unclear.

Consequently, we assumed that the dietary addition of rutin could improve the growth performance, content or composition of PUFAs, anti-oxidative capacity, and meat quality of Qingyuan partridge chicken. To test this hypothesis, we conducted this study to examine the influences of rutin supplementation on meat quality, muscle FA composition, and the antioxidant capacity of Qingyuan partridge chicken. Our study may establish a theoretical foundation for the potential application of rutin in the production of Qingyuan partridge chicken as well as in the broader broiler industry.

## 2. Materials and Methods

### 2.1. Animals, Experimental Design, and Diets

One hundred and eighty healthy female Qingyuan partridge chickens (17 weeks old) were subjected to a randomized assignment into three groups, identified as the control, R200, and R400 groups, with respective supplementation of 0, 200, and 400 mg/kg rutin. There were 6 replicates in each group, with each replicate consisting of 10 chickens. Rutin (purity, ≥95%) was ordered from Shanghai Aladdin Biotechnology Co., Ltd. (Shanghai, China). The control diet was formulated in accordance with the recommendations of the nutrient requirements of yellow chickens (Ministry of Agriculture of China, 2020) to satisfy their nutritional requirements (Table 1). All chickens were raised in 3-tier battery cages with 2 chickens per cage (cage size: 78 cm × 40 cm × 39 cm). Ad libitum access to feed and drinking water was provided to the chickens throughout the experimental duration, comprising a pre-feeding period of 5 days and a formal period of 30 days. The housing temperature and relative humidity were maintained at 16–20 °C and 50–60%, respectively. Chickens were reared under a 16:8 light/dark lighting regimen (approximately 20 lx from 5:00 a.m. to 10:00 p.m.) using white LED light. The growth performance was assessed by measuring the average daily gain (ADG), average daily feed intake (ADFI), and feed-to-gain ratio (F/G). The birds were weighed by replicate on day 1 (initial body weight (BW)) and day 35 (final BW) after 8 h of starvation, and BW gain was determined by ascertaining the difference between the initial and final weights obtained on the aforementioned days. The feed intake of birds was recorded for each replicate weekly. The F/G was computed as feed intake divided by BW gain in each replicate.

### 2.2. Slaughtering, Sample Collection, and Preparation

After the 35-day feeding trial, two chickens from each replicate group were randomly chosen (a total of 12 broiler chickens per group) and slaughtered after approximately 8 h of fasting. Approximately 10 mL of fasting blood was drawn from the wing vein, kept undisturbed at room temperature for 30 min, and subsequently subjected to centrifugation at 3000 rpm for 10 min at 4 °C. One mL aliquot of serum was collected and preserved at a temperature of −80 °C for subsequent analytical procedures. Following the blood collection procedure, the chickens were hanged and then killed by severing the carotid artery. After defeathering and weighing, the carcasses were cut open to remove the organs, including the esophagus, trachea, crop, pancreas, intestinal tract, spleen, and reproductive organs. The semi-eviscerated percentage, eviscerated percentage, breast muscle percentage, thigh muscle percentage, and abdominal fat percentage were measured according to a previous study [12]. The left-side breast muscle was collected for the determination of meat color and muscle pH. In addition, the right-side breast muscle (about 50.0 g) was collected to measure dripping loss and shear force value. About 50 g of the remaining right-side breast muscle was collected and immediately immersed in liquid-N_2_ before being preserved at −80 °C for later analysis of IMF, inosine monophosphate (IMP), FA composition, antioxidant capacity, and gene expression.

### 2.3. Meat Quality Assay

The pH and color (L*, a*, and b* value) of breast muscle were assessed at 45 min and 24 h postmortem using a hand-held pH meter (model HI 9024C HANNA Instruments, Ltd., Beijing, China) and a Chroma meter (model CR-410 Konica Minolta Sensing Ins., Osaka, Japan), respectively. The measurement of drip loss and shear force of breast muscle was respectively performed in accordance with the method described by Honikel [13] and Yu et al. [14].

### 2.4. Analysis of Muscle Chemical Composition and Serum Biochemical Indexes

The determination of IMF content in breast muscle was carried out according to a previous study [15]. The IMP level in muscle samples was determined in accordance with the method described by Li et al. [16]. For the analysis of the FA profile in breast muscle, total lipids of the freeze-dried samples were isolated with chloroform–methanol (1:1, *v*/*v*), according to Folch et al. [17]. The concentration of FA methyl esters in breast muscle was determined with the method described by Yu et al. [18]. The determination of FA methyl esters was carried out on the Agilent 7890B gas chromatographer system, as described previously [19]. The FA content was presented as the percentage of total FAs (%). Additionally, in order to indirectly assess the desaturase activity within breast muscle, desaturation indexes were calculated by determining the proportion of product to a precursor for the individual FA [20].

The contents of total protein (TP), albumin (ALB), creatinine (CRE), urea nitrogen (BUN), uric acid (UA), glucose (GLU), TG, TC, high-density lipoprotein (HDL), and low-density lipoprotein (LDL) in serum were measured using a Selectra ProXL automatic biochemical analyzer system (Vital Scientific N.V., Dieren, The Netherlands) with specific kit (Zhongsheng Beikong Biotechnology Co., Ltd., Beijing, China).

### 2.5. Antioxidant Capacity

The total antioxidant capacity (T-AOC), malondialdehyde (MDA) content, and activities of total superoxide dismutase (T-SOD), catalase (CAT), and glutathione peroxidase (GSH-Px) in serum and breast muscle were determined using specific kits (Nanjing Jiancheng Bioengineering Institute, Nanjing, China) in accordance with the manufacturer’s instructions.

### 2.6. Quantitative Real-Time PCR (qRT-PCR) Analysis

Total RNA from breast muscle was extracted using TRIzol reagent (Takara Biotechnology, Tokyo, Japan). The purity and concentration of the obtained total RNA were determined with a NanoDrop-1000 spectrophotometer (Thermo Fisher Scientific Inc., Waltham, MA, USA). Subsequently, the cDNA was synthesized in accordance with the manufacturer’s instructions, utilizing a cDNA Synthesis Kit (Takara Biotechnology). The cDNA samples were used to measure the mRNA expression by qRT-PCR on the CFX Connect detection system (Bio-Rad Laboratories, Hercules, CA, USA). The qRT-PCR was performed using the TB Green™ Premix Ex Taq™ (Takara Biotechnology), and the specific primers used for qRT-PCR (Table 2) were ordered from Sangon Biotech (Shanghai, China). The qRT-PCR reaction mixture and procedure were performed as previously described [14]. The expression levels of each target gene were normalized using the internal control gene β-actin. The fold expression of each target gene was determined according to the 2^−ΔΔCt^ method.

### 2.7. Statistical Analysis

The data analysis in this study was conducted using the SPSS (v. 20.0) software package (IBM Corporation, Armonk, NY, USA). The assessment of the growth performance was performed based on the following parameters initial BW, final BW, ADG, ADFI, and F/G, which were analyzed using replicate as the experimental unit, while other parameters were calculated using the average of two chickens slaughtered in each replicate as the experimental unit. Differences among groups were examined by conducting a one-way analysis of variance (ANOVA) followed by Tukey’s post hoc test. Statistical significance was set at α = 0.05 level. Multivariate analysis of FA content in breast muscle was performed using SIMCA 14.1 Software (Umetrics AB, Umea, Sweden). The model of partial least squares discriminant analysis (PLS-DA) was applied to discriminate the fatty acid profiles among groups.

## 3. Results

### 3.1. Growth Performance and Carcass Traits

The growth performance and carcass traits of Qingyuan partridge chickens are shown in Table 3. Concerning growth performance, the three groups under investigation exhibited no significant discrepancies in initial BW, final BW, ADG, ADFI, and F/G (*p* > 0.05). Regarding carcass traits, no differences were found among the three groups in the carcass yield, semi-eviscerated yield, eviscerated yield, thigh muscle yield, and abdominal fat yield (*p* > 0.05). However, the yield of breast muscle in the R200 group was significantly higher than that in the control group (*p* < 0.05).

### 3.2. Meat Quality

The effects of rutin supplementation on meat quality are shown in Table 4. Dietary rutin supplementation showed no significant difference in pH_45min_, pH_24h_, color parameters, shear force, and IMP content of the breast meat among the three groups (*p* > 0.05). The drip loss of Qingyuan partridge chicken breast meat was significantly decreased in response to rutin supplementation (*p* = 0.005), with the minimum observed in the R200 group. However, the IMF of breast muscle in the R400 group was significantly increased compared with the control group (*p* < 0.05).

### 3.3. Serum Biochemical Indexes

As shown in Table 5, the contents of serum GLU in the R200 and R400 groups were significantly decreased in comparison to the control group (*p* < 0.05). Additionally, rutin treatment significantly decreased the levels of TG and TC in the serum of Qingyuan partridge chickens (*p* < 0.05). In contrast, the level of serum HDL in the R400 group was significantly higher than that in the control group (*p* < 0.05), while the supplementation with 200 mg/kg rutin had no significant effect on serum HDL (*p* > 0.05). Dietary rutin supplementation tended to decrease the content of LDL in serum (*p* = 0.068). Moreover, adding rutin to the diet of Qingyuan partridge chickens had no significant effect on the serum TP, ALB, CRE, BUN, and UA (*p* > 0.05).

### 3.4. Fatty Acid Profile of Breast Muscle

**Figure 1:**
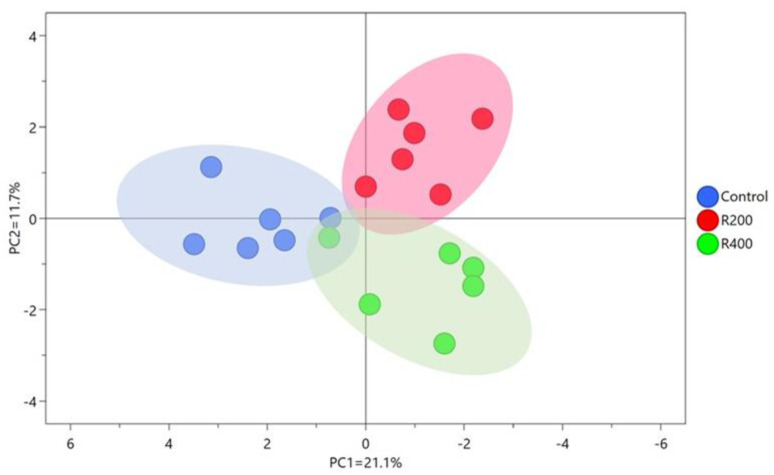
PLS-DA score plot of FA profile, based on the concentration of FAs in breast muscle of Qingyuan partridge chickens. Each point represents a replicate in the scatter plots. Control, R200, and R400 mean the groups supplemented with 0, 200, and 400 mg/kg rutin, respectively.

As shown by the PLS-DA model (Figure 1), there was a clear separation in fatty acid profile among the groups. The specific changes in fatty acids in the breast muscle of Qingyuan partridge chickens are shown in Table 6. All the saturated fatty acids (SFAs) detected in breast muscle, except for the C10:0, were not significantly altered by the rutin supplementation (*p* > 0.05). For the monounsaturated fatty acids (MUFAs), dietary supplementation with 200 and 400 mg/kg rutin significantly decreased the content of C16:1 (*p* < 0.05) in breast muscle. Although the supplementation with rutin did not affect the concentration of individual *n*-6 PUFAs (including C18:2 (*n* − 6), C18:3 (*n* − 6), C20:2 (*n* − 6), C20:3 (*n* − 6), and C20:4 (*n* − 6)), the R200 group showed an increasing trend in the sum of *n*-6 PUFAs in comparison to the other two groups (*p* = 0.092). Furthermore, the concentrations of C22:6 (*n* − 3) and the sum of *n*-3 PUFAs were markedly elevated in the R200 group as compared to the control group (*p* < 0.05). Additionally, significantly higher levels of the sum PUFAs and ratio of PUFAs/SFAs were observed in the R200 group compared to the control group (*p* < 0.05). However, the ratio of *n*-6/*n*-3 PUFAs in the R200 group was significantly reduced compared to the control group (*p* < 0.05).

As shown in Table 6, the activities of Δ9 (16:1 (*n* − 7)/16:0) in the rutin-treated groups were significantly reduced in comparison to the control treatment (*p* < 0.05). The treatment with 400 mg/kg rutin showed an increasing trend in the activity of Δ6 (20:3 (*n* − 6)/18:2 (*n* − 6)) compared with that in the other two treatments (*p* = 0.084). Furthermore, the R200 group had higher activity of Δ5 + Δ6 (22:6 (*n* − 3)/18:3 (*n* − 3)) as compared to the control group (*p* < 0.05). No significant differences in the activities of Δ9 (18:1 (*n* − 9)/18:0), Δ5 (20:4 (*n* − 6)/20:3 (*n* − 6)), and Δ5 + Δ6 (20:4 (*n* − 6)/18:2 (*n* − 6)) were observed across the three groups (*p* > 0.05).

### 3.5. Antioxidant Capacity

The antioxidant capacity of different tissues is shown in Table 7. In serum, the MDA content exhibited a significant reduction in response to the rutin treatment (*p* < 0.05), with a minimum observed in the R200 group. However, rutin supplementation significantly elevated the contents of CAT, T-SOD, and T-AOC (*p* < 0.05). Notably, in breast muscle, the MDA content exhibited a linear decrease upon incremental intake of rutin supplementation (*p* < 0.05). In comparison to the control group, the content of CAT in the R200 group was significantly elevated (*p* < 0.05). In addition, the contents of T-AOC both in the R200 and R400 groups were significantly higher than that in the control group (*p* < 0.05).

### 3.6. Changes in mRNA Expression of Genes Associated with Lipid Metabolism and Antioxidant Capacity in Breast Muscle

**Figure 2:**
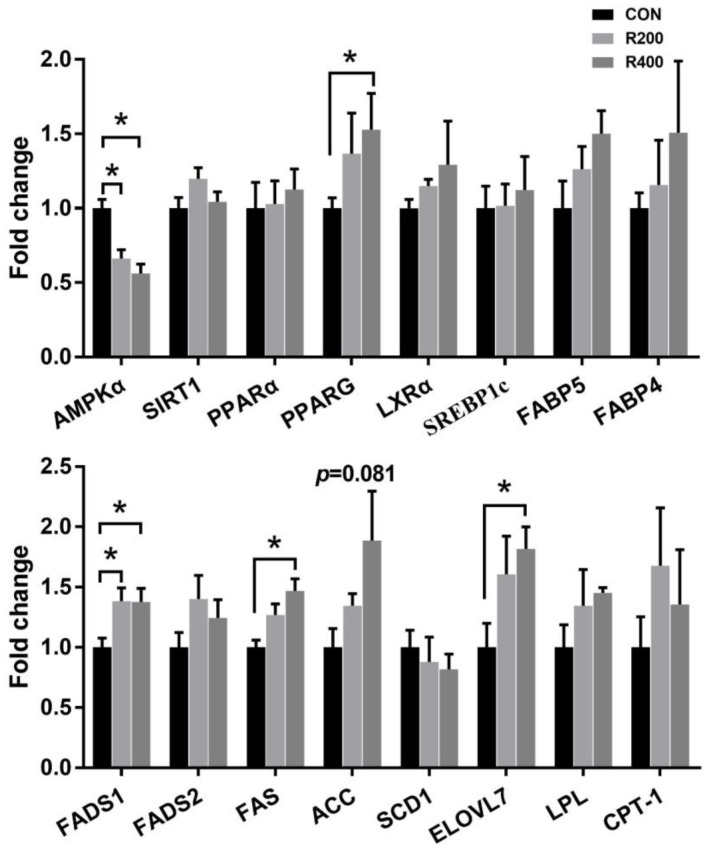
Effects of rutin supplementation on the relative expression of the genes associated with lipid metabolism in breast muscle of Qingyuan partridge chickens. Values are presented as the means ± SEM (*n* = 6). Asterisk means statistical significance between the treatment groups at α = 0.05 level. CON, R200, and R400 mean the groups supplemented with 0, 200, and 400 mg/kg rutin, respectively. ACC, acetyl-CoA carboxylase; AMPKα, adenosine monophosphate-activated protein kinase alpha; CPT-1, carnitine palmitoyltransferase-1; ELOVL7, long-chain fatty acid elongase 7; FABP4, fatty acid binding protein 4; FABP5, fatty acid binding protein 5; FADS1, fatty acid desaturase 1; FADS2, fatty acid desaturase 2; FAS, fatty acid synthase; LPL, lipoprotein lipase; LXRα, liver X receptor alpha; PPARα, peroxisome proliferator-activated receptor alpha; PPARG, peroxisome proliferator-activated receptor gamma; SCD1, stearyl coenzyme A decarboxylase 1; SIRT1, sirtuin 1; SREBP1c, sterol regulatory element binding protein 1c.

To further investigate the mechanisms involved in the regulation of IMF and *n*-3 PUFA deposition by treatment with rutin, the relative mRNA expression levels of genes associated with lipid metabolism in pectoralis muscle (Figure 2) were measured by qRT-PCR. Rutin supplementation significantly decreased the mRNA expression levels of adenosine monophosphate-activated protein kinase alpha (*AMPKα*) (*p* < 0.05). Nevertheless, treatment with rutin significantly elevated the mRNA expression levels of FA desaturase 1 (*FADS1*) (*p* < 0.05). Moreover, the peroxisome proliferator-activated receptor gamma (*PPARG*), FA synthase (*FAS*), and FA elongase 7 (*ELOVL7*) expressions in the R400 group were significantly upregulated as compared to the control group (*p* < 0.05). Additionally, rutin supplementation tended to upregulate the expression of acetyl-CoA carboxylase (*ACC*) (*p* = 0.081). The three groups exhibited no significant differences in the mRNA expression levels of *SIRT1*, *PPARα*, *LXRα*, *SREBP1c*, *FABP4*, *FABP5*, *FADS2*, *SCD1*, *LPL,* and *CPT-1* (*p* > 0.05).

**Figure 3:**
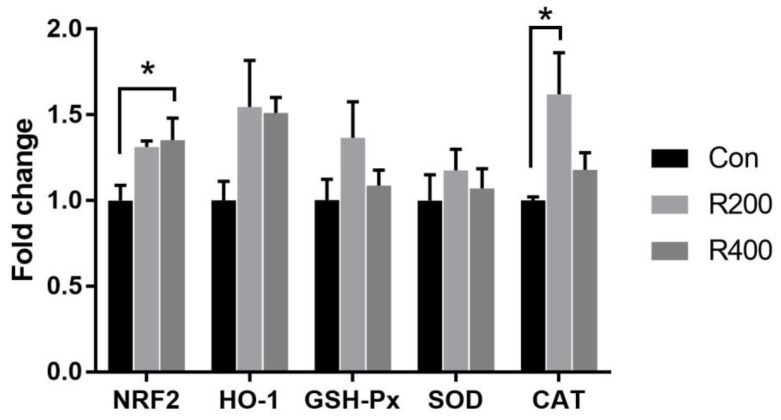
Effects of rutin supplementation on the relative expression of antioxidant-related genes in breast muscle of Qingyuan partridge chickens. Values are expressed as the means ± SEM (*n* = 6). Asterisk means statistical significance between the treatment groups at α = 0.05 level. Con, R200, and R400 mean the groups supplemented with 0, 200, and 400 mg/kg rutin, respectively. CAT, catalase; GSH-Px, glutathione peroxidase; HO-1, heme oxygenase-1; *NRF2*, nuclear factor erythroid 2-related factor 2; SOD, superoxide dismutase.

The mRNA expression levels of antioxidant-related genes in the pectoralis muscle are shown in Figure 3. In particular, the relative mRNA expression of nuclear factor erythroid 2–related factor 2 (*NRF2*) in the R400 group was significantly elevated as compared to the control group (*p* < 0.05). The catalase (*CAT*) mRNA expression in the R200 group was significantly upregulated compared with that in the control group (*p* < 0.05). Dietary rutin supplementation did not significantly alter the relative mRNA expressions of *HO-1*, *GSH-Px,* and *SOD* (*p* > 0.05).

## 4. Discussion

Consumer awareness of the link between wholesomeness and balanced nutritional food is increasing, as well as the need for a product with the right taste and quality profile to meet their needs. Flavonoids are valuable feed additives to improve gut health or meat quality in poultry. Rutin, one of the cheapest flavonoids, has a variety of biological functions, such as antioxidant, antidiabetes, and antidyslipidemia activity [10,21,22], and may have regulatory effects on lipid metabolism, fatty acid synthesis, and meat quality of Chinese indigenous, yellow-feathered broiler. In this study, rutin supplementation dramatically increased the breast muscle yield, IMF, HDL, *n*-3 PUFAs, and antioxidant capacity but decreased the drip loss, TG levels, TC levels, and the proportion of *n*-6/*n*-3 PUFAs of Qingyuan partridge chickens. Additionally, these alterations were linked to the upregulated expression of lipid metabolism-related and antioxidant-related genes. These findings highlight the improvement of meat quality, antioxidant capacity, and especially, the FA profile in Qingyuan partridge chickens, suggesting that rutin supplementation contributes to the increased nutritional value and taste of the meat of Qingyuan partridge chickens.

### 4.1. Dietary Rutin Supplementation Did Not Alter the Growth Performance of Qingyuan Partridge Chickens

Recently, the utilization of flavonoids as natural and safe feed additives capable of enhancing growth and meat quality has emerged as an effective approach to livestock production. Emerging evidence shows that rutin could facilitate the growth performance of broilers. The inclusion of 1000 g/kg rutin in diets increased body weight gain and decreased F/G of Rose-broilers in the trial duration (days 1–42) [8]. Likewise, dietary supplementation with 500 mg/kg rutin increased BW, ADFI, and ADG and decreased F/G of Arbor Acres broilers in the trial duration (days 1–42) [9]. Inconsistent with the studies above, the present findings showed that rutin supplementation had no effect on the growth performance of Qingyuan partridge chickens. It has been reported that the favorable effect of polyphenol-rich grapes on broiler growth was likely attributed to improved gut morphology and function [23]. Moreover, the upregulated levels of hormones, including growth hormone and insulin-like growth factor 1, possibly accounted for the promotion of broiler growth by alfalfa flavonoid supplementation [6]. Similarly, the potential mechanism by which rutin supplementation improved the growth performance of broilers was due to the promotion of intestinal barrier function and the upregulation of growth hormone levels [9]. Therefore, it is possible that the discrepant effect of rutin on growth performance is on account of the variations in the developmental stages of the chicken subjects under investigation, as indirectly evidenced by the findings demonstrating that a diet supplemented with 250 mg rutin/kg significantly improved the growth performance of broiler in days 1–21 while having no significant differences in growth performance in days 22–42 [9]. However, additional studies are required regarding the mechanisms underlying the enhancement of growth performance by rutin supplementation in chicken.

### 4.2. Dietary Rutin Supplementation Altered the Meat Quality of Qingyuan Partridge Chickens

Drip loss is a vital indicator for judging meat quality, which is tightly correlated with the physical structure, flavor, juiciness, and color of meat. Unfavorably high drip loss would not only induce the loss of soluble flavor substance in meat but would also have a detrimental impact on the sensory quality of the meat end-product and consumer acceptance [24]. Our results revealed that drip loss in breast muscle was markedly reduced after the dietary inclusion of rutin. Likewise, dietary supplementation with alfalfa flavonoids was reported to have a significant decrease in breast-muscle drip loss of broilers [6]. Consistent with the results above, dietary supplementation with hesperidin reduced breast-muscle drip loss of broilers [25]. Lipid peroxidation can change the membrane structure and functions of muscle cells and impair their water-holding capacity [26]. Moreover, secondary products of lipid oxidation could accelerate the oxidation of proteins and change their stability or peptide structure, resulting in substantial loss of biological function and water-holding capacity [27,28]. Thus, the increased antioxidant capacity in broilers by supplementation with flavonoids probably accounts for the decreased drip loss [6,25].

The IMF content is also thought to be a critical factor in determining the nutritional value, tenderness, flavor, and juiciness of the meat. Our findings also indicated that dietary rutin supplementation significantly increased IMF content in breast muscle. Similarly, dietary supplementation with flavones of sea buckthorn fruits was reported to significantly increase the IMF level in breast and thigh muscles, presumably as a result of the increased levels of insulin and leptin in serum [29]. However, dietary supplementation with soybean isoflavone was not found to alter the IMF concentration of breast muscle in broilers [7], and hesperidin supplementation resulted in no significant change in the IMF concentration of the *Longissimus thoracis* muscle of lambs [30]. These inconsistencies may be relevant to the differences in experimental animals, the kinds of flavonoids used, and the duration of the experiment. The IMF could retain higher water content by altering the microstructure of meat, which facilitates the juiciness and tenderness of meat [31]. Our results have provided potential evidence that rutin supplementation significantly increased IMF while simultaneously reducing drip loss. In addition, IMF also contributes to the flavor profile of meat due to its high content of UFAs, which act as crucial precursors for the formation of desirable flavor compounds in meat [32]. Accordingly, the augmented content of IMF, which, in turn, could potentially enhance the meat’s sensory attributes, such as tenderness, juiciness, and flavor, may account for the improved meat quality by dietary rutin supplementation. However, further investigations are needed to elucidate the mechanisms involved in the regulation of the IMF content by rutin supplementation.

To the best of our knowledge, the present study, for the first time, investigates the influence of rutin supplementation on the meat quality of chickens. Our results suggested that the addition of rutin to the diet improved the nutritional value, juiciness, and water retention ability of the meat of Qingyuan partridge chickens.

### 4.3. Dietary Rutin Supplementation Altered the Fatty Acid Profile and Lipid Metabolism of Qingyuan Partridge Chickens

The FA profile of IMF is one of the most critical indexes closely correlated with the flavor and nutritional value of the meat [33]. Meat with a healthier FA profile is of considerable interest to the meat industry and consumers. PUFAs, particularly *n*-3 PUFAs, including α-linolenic acid (ALA, C18:3*n* − 3) and DHA, exert a central role in promoting the formation of meat flavor substances and maintaining human health [34]. However, many organisms, including humans, lack the enzymes to synthesize ALA de novo, which is a crucial precursor for DHA; therefore, these organisms must obtain ALA from the diet [35]. DHA is mainly obtained through dietary sources or biosynthesis from ALA. The conversion of ALA to DHA involves a series of complex elongation and desaturation processes, in which Δ5 and Δ6 desaturases encoded by FA desaturase 1 and 2 (*FADS1* and *FADS2*) genes are the rate-limiting enzymes [36]. It has been shown that the administration of flavonoid supplements (anthocyanin extracted from corn) increased the concentrations of DHA and *n*-3 PUFAs in the plasma of rats [37]. However, Gallegos et al. [38] reported that dietary supplementation with anthocyanin extracted from purple corn did not affect hepatic EPA and DHA concentrations in rats. In chicken, the concentrations of DHA and *n*-3 PUFAs in the hen liver were considerably increased by genistein supplementation [39]. Furthermore, a linseed oil (ALA-rich)-enriched diet supplemented with soybean isoflavone significantly increased the EPA and DHA contents in breast muscle in comparison to a linseed oil diet alone, accompanied with higher *FADS2* gene expression [7]. In accordance with the above two reports, we also found that rutin dietary inclusion increased the content of DHA and *n*-3 PUFAs in breast muscle, thereby improving fatty acid composition in chickens. In addition, higher *FADS1* mRNA levels and the product-to-precursor ratio of Δ5 and Δ6 activity were observed in chickens with rutin supplementation. Additionally, flavonoids, including quercetin, daidzein, and genistein, could increase the mRNA levels of Δ5 or Δ6 desaturases in HepG2 cells [40]. Thus, the increased level of *n* − 3 PUFAs in the present study may result from the increased activity and mRNA expression of Δ5 and Δ6 desaturases. Noteworthy, to date, the research on chickens seems more consistent than that on other organisms, including rodents and humans. On the other hand, PUFA-rich chickens contain a greater proportion of double-bonded fatty acids, which are vulnerable to oxidative attack [41]. The increased antioxidant capacity could provide oxidative-proof protection for PUFAs and possibly contribute to the accumulation of PUFAs. However, further investigations are needed to establish the underlying mechanisms.

The content of lipid metabolism-related products can be used as an indicator to assess the lipid metabolism function of the body and is tightly linked with the growth and development of animals and their health states [42]. The TG and TC are important fractions of blood fat, and their contents reflect the fat utilization rate. LDL enriches cholesterol, and a high content of LDL can cause atherosclerosis. HDL could carry cholesterol from peripheral tissue to the liver to synthesize bile acid, which contributes to reducing the formation of atherosclerosis [43]. It has been reported that oral rutin could decrease serum TC and TG contents and increase the serum HDL content in rats [44]. Furthermore, dietary rutin supplementation was found to decrease the contents of TC, TG, and LDL in the serum of broilers [8]. These studies are in accordance with our finding that dietary rutin supplementation significantly decreased the serum contents of TC and TG but increased the serum HDL content. Flavonoids could be reciprocal with cholesterol and suppress the intestinal absorption of endogenous and exogenous cholesterol by forming insoluble complexes [45]. In parallel, rutin reduced TG synthesis through the reduction in lipid synthesis and acceleration of FA catabolism in the liver [46]. Thus, a possible explanation for the improvement of the homeostasis of blood lipids is that rutin could inhibit the synthesis of lipid and intestinal absorption of cholesterol and accelerate the lipid decomposition of cells in the liver and the reverse transport of cholesterol.

TGs, as the primary constituent of IMF, are crucial for the manipulation of energy metabolism in muscle. The metabolic processes involved in energy utilization and lipid storage in muscular tissue are inseparable from the biosynthesis and deposition of TGs [47]. As a key molecule in maintaining energy balance, AMPK regulates lipid accumulation and gene transcription [48]. AMPK increases FA oxidation, reduces FA synthesis, and improves lipid homeostasis through activation of Carnitine palmitoyl transterase-1 (CPTl) and PPARα and suppression of ACC in the liver [49]. It has been reported that the IMF of the *biceps femoris* muscle in dry-lot feeding lambs was increased by downregulating the expression level of AMPK and upregulating the expression level of ACC compared to those in grazing lambs [50]. Consistent, in part, with the above study, our study showed that rutin supplementation downregulated mRNA expression levels of *AMPK* and tended to upregulate mRNA expression levels of *ACC* in breast muscle, which is partially responsible for the increased lipid deposition. PPARs are AMPK downstream targets, and AMPK could regulate body fat deposition by decreasing mRNA expression of *PPARG* [51]. PPARG is an essential manipulator that regulates adipogenesis and glucose homeostasis. In mice, rutin was reported to upregulate the expression level of *PPARG* in skeletal muscles [52]. In this study, rutin supplementation upregulated the expression level of *PPARG* in breast muscle. Thus, the AMPK/PPARG signaling pathway may regulate the IMF content in breast muscle. FAS represents a critical regulatory component of the biosynthetic pathway of FA, primarily mediating the ultimate step of FA synthesis in muscular tissue [53]. In contrast to the previous studies [51,54], indicating that rutin inhibited the expression and transcription of *FAS* in hepatocytes, our results indicated that the mRNA expression of *FAS* was upregulated by rutin supplementation. These controversial results might be due to the different species of animals and tissues used for the measurement. Long-chain fatty acids (LCFAs) and very long-chain fatty acids (VLCFAs) are important participants in the synthesis of TGs and lipid metabolism. The elongation of the very long-chain fatty acid (ELOVL) protein family is necessary for the synthesis of LCFAs and VLCFAs [55]. In particular, *ELOVL7*, a newly discovered ELOVL protein family member, evokes lipid accumulation in differentiated adipocytes [56]. *ELOVL7* was found to be one of the overexpressed key genes associated with adipogenesis and lipogenesis in the breast muscle of animals in the group with higher TG content [47]. Therefore, the increased mRNA expression of *ELOVL7* is potentially responsible for the rutin treatment-induced increased IMF in breast muscle. Overall, these findings indicate that rutin supplementation changed the FA profile and lipid metabolism in Qingyuan partridge chickens by manipulating the lipid metabolism-related gene expression.

### 4.4. Dietary Rutin Supplementation Altered the Antioxidant Capacity of Qingyuan Partridge Chickens

The antioxidant capacity is positively linked with the body’s health and meat quality [57]. The MDA content is considered an index for the degree of lipid peroxidation [58]. SOD, GSH-Px, and CAT are the main antioxidant enzymes in the organism, which can efficiently scavenge free radicals. Furthermore, T-AOC broadly reflects the antioxidant capacity of living organisms [59]. Hassan et al. [8] showed that the activities of anti-oxidase (SOD, GSH-Px, and CAT) and the MDA content in the serum of broilers were increased and decreased by rutin supplementation, respectively. Similarly, dietary rutin supplementation has been found to increase the T-AOC and T-SOD activities in the jejunal mucosa of broilers [9]. Our findings also indicated that rutin supplementation prominently elevated the T-AOC and CAT activities but decreased the MDA content in serum and breast muscle. The activities of antioxidant enzymes partly depend on their gene expression levels, which have been shown to be regulated by *NRF2* [60]. In this study, we further revealed that rutin supplementation increased the mRNA levels of *NRF2* and *CAT* in breast muscle. Rutin, which is regarded as a powerful antioxidant, effectively inhibited lipid peroxidation and directly scavenged the generated ROS, such as hydroxyl radicals, H_2_O_2,_ and superoxide anions [61]. Rutin also promoted the production of GSH-Px and SOD by activating the *NRF2* signaling pathway, which led to the improvement of the scavenging of free radicals and maintenance of the body’s redox balance [62]. Therefore, dietary rutin supplementation can enhance the antioxidant capacity and alleviate oxidative stress, ultimately having a positive effect on the health and meat quality of chickens.

## 5. Conclusions

In summary, this study demonstrated that the dietary inclusion of rutin improved the meat quality of Qingyuan partridge chickens by elevating the levels of IMF and *n*-3 PUFAs, and antioxidant capacity and decreasing the drip loss and the proportion of *n*-6/*n*-3 PUFA. Additionally, these alterations were linked to the altered expression levels of lipid metabolism-related and antioxidant-related genes. These findings highlight the beneficial impact of rutin on meat quality, antioxidant capacity, and, in particular, the fatty acid profile in Qingyuan partridge chickens, suggesting that rutin supplementation leads to a more nutritional, tastier, and healthier meat in Qingyuan partridge chickens.

## Figures and Tables

**Table 1:** Ingredients and nutritional composition of the control diet.

Items	Content
Ingredients, %	
Yellow maize	78.50
Soybean meal	12.00
Corn gluten meal	4.00
Soybean oil	1.40
L-Lys HCl	0.30
DL-Methionine	0.10
CaHPO_4_	1.20
Limestone	1.20
NaCl	0.30
Vitamin and mineral premix ^1^	1.00
Total	100.00
Calculated nutrient composition ^2^, %	
Metabolizable energy, MJ/kg	12.91
Lysine	0.82
Methionine	0.37
Ca	0.77
Total phosphorus	0.52
Available phosphorus	0.28
FA (% total FA)	
C14:0	0.10
C16:0	10.66
C18:0	2.00
ΣSFA	12.76
C16:1	0.36
C18:1	25.19
ΣMUFA	25.55
C18:2	54.03
C18:3	1.86
ΣPUFA	55.88
Analyzed composition, %	
Crude protein	14.87

^1^ Provided per kilogram of complete diet: vitamin A, 3600 IU; vitamin D, 1650 IU; vitamin E, 16.5 mg; vitamin K3, 1.65 mg; vitamin B1, 1.5 mg; vitamin B2, 4.5 mg; vitamin B6, 3 mg; nicotinic acid, 30 mg; pantothenic acid, 9 mg; folic acid, 0.75 mg; vitamin B12, 0.015 mg; biotin, 0.075 mg; Cu, 7.2 mg; Fe, 72 mg; Zn, 66 mg; Mn, 78 mg; I, 0.42 mg; Se, 0.29 mg. ^2^ Values were calculated using the feed composition and nutritive values provided by Feed Database in China (2020), except for the crude protein.

**Table 2:** Primers used for qRT-PCR assay.

Gene	Forward Primer (5′–3′)	Reverse Primer (5′–3′)	Sequence	Product Size (bp)
ACC	TTGTGGCACAGAAGAGGGAA	GTTGGCACATGGAATGGCAG	NM_205505.1	161
AMPKα	GGGACCTGAAACCAGAGAACG	ACAGAGGAGGGCATAGAGGATG	NM001039605.1	215
CAT	GGTTCGGTGGGGTTGTCTTT	CACCAGTGGTCAAGGCATCT	NM_0,010,31215.1	213
CPT-1	ACAGCGAATGAAAGCAGGGT	GCCATGGCTAAGGTTTTCGT	NM_001012898.1	93
ELOVL7	GGCCACTCATGTCTTCACCT	TGTAGGCGACCTCGAGTAGT	NM_001197310.1	245
FABP4	GGGGTTTGCTACCAGGAAGATG	CATTCCACCAGCAGGTTCCC	NM_204290.1	276
FABP5	AAATGGGAAGCATGGCGAAAC	TCACATTCCACCACCAACTGT	NM_001006346.1	265
FADS1	GGAAACAGTGGGTGGACCT	AGATGAAGCCCCAGGATACC	XM_421052.2	103
FADS2	AATTGAGCACCACCTGTTCC	TGGCACATAACGACTTCACC	NM_001160428.2	80
FAS	AGAGGCTTTGAAGCTCGGAC	GGTGCCTGAATACTTGGGCT	NM_205155.2	127
GSH-Px	GACCAACCCGCAGTACATCA	GAGGTGCGGGCTTTCCTTTA	NM_0,012,77853.1	204
HO-1	GGTCCCGAATGAATGCCCTTG	ACCGTTCTCCTGGCTCTTGG	HM237181.1	137
LXRα	GTCCCTGACCCTAATAACCG	TCTCGGAAGCCAGGTAGTTGCT	AF492498.1	119
NRF2	GATGTCACCCTGCCCTTAG	CTGCCACCATGTTATTCC	NM_205,117.1	216
PPARα	AGTAAGCTCTCAGAAACTTTGTTG	AAGGTTGAAACAGAAGCCGC	NM_001001464.1	162
PPARG	CCAGCGACATCGACCAGTTA	TCCCATCCTTAAAGAGTTCA	NM_001001460.1	182
SCD1	CATGGGCCATTCTGTGCTT	GGCCATGGAGTTTGCAATAG	NP_990221.2	131
SOD	CCGGCTTGTCTGATGGAGAT	TGCATCTTTTGGTCCACCGT	NM_205,064.1	125
SIRT1	GATCAGCAAAAGGCTGGATGGT	ACGAGCCGCTTTCGCTACTAC	NM_001004767	143
SREBP-1c	GCCCTCTGTGCCTTTGTCTTC	ACTCAGCCATGATGCTTCTTC	XM_046927256.1	130
β-Actin	TGCTGTGTTCCCATCTATCG	TTGGTGACAATACCGTGTTCA	NM_205,518.1	150

**Table 3:** Effect of rutin supplementation on growth performance and carcass traits of Qingyuan partridge chickens.

Items	Rutin Supplementation, mg/kg			*p*-Value
	0	200	400	
Growth Performance				
Initial BW, g	1741.62 ± 5.59	1739.64 ± 11.11	1739.02 ± 4.35	0.828
Final BW, g	2238.13 ± 112.72	2288.44 ± 73.10	2296.25 ± 53.36	0.444
ADG, g/d	14.19 ± 3.13	15.68 ± 1.96	15.92 ± 1.61	0.398
ADFI, g/d	117.03 ± 10.71	118.74 ± 12.67	117.71 ± 11.33	0.968
F/G	8.51 ± 1.46	7.61 ± 0.61	7.40 ± 0.43	0.132
Carcass Traits, %				
Carcass yield	91.02 ± 0.83	91.00 ± 0.93	90.82 ± 0.39	0.881
Semi-eviscerated yield	79.73 ± 2.40	80.28 ± 1.52	79.87 ± 1.49	0.867
Eviscerated yield	68.74 ± 2.42	70.44 ± 0.86	69.96 ± 1.13	0.206
Breast muscle yield	12.25 ± 0.98 ^b^	13.42 ± 0.57 ^a^	13.33 ± 0.98 ^ab^	0.038
Thigh muscle yield	17.26 ± 0.96	16.54 ± 1.66	16.53 ± 0.66	0.484
Abdominal fat yield	6.08 ± 0.27	5.57 ± 0.33	6.01 ± 0.67	0.151

^a,b^ within a row with different superscripts mean significant differences at α = 0.05 level. Values are presented as means ± standard deviation (*n* = 6). BW, body weight; ADG, average daily gain; ADFI, average daily feed intake; F/G, feed-to-gain ratio.

**Table 4:** Effect of different levels of rutin on breast meat quality of Qingyuan partridge chickens.

Items	Rutin Supplementation, mg/kg			*p*-Value
	0	200	400	
pH_45min_	6.14 ± 0.11	6.08 ± 0.32	6.17 ± 0.33	0.813
pH_24h_	5.69 ± 0.05	5.70 ± 0.07	5.72 ± 0.06	0.818
L*_45min_	58.63 ± 1.67	59.53 ± 1.87	59.74 ± 3.66	0.731
a*_45min_	13.00 ± 1.12	12.24 ± 1.30	12.81 ± 1.14	0.527
b*_45min_	19.27 ± 1.68	19.98 ± 2.61	18.18 ± 1.73	0.336
L*_24h_	65.48 ± 1.15	64.29 ± 2.19	64.60 ± 1.25	0.431
a*_24h_	10.52 ± 0.52	10.84 ± 1.14	11.16 ± 1.95	0.718
b*_24h_	21.40 ± 2.20	21.92 ± 2.79	20.56 ± 1.19	0.561
Drip loss, %	2.11 ± 0.20 ^a^	1.79 ± 0.12 ^b^	1.92 ± 0.08 ^b^	0.005
Shear force, N	23.67 ± 1.91	23.86 ± 2.22	25.95 ± 2.05	0.140
IMF %	0.21 ± 0.02 ^b^	0.32 ± 0.13 ^ab^	0.37 ± 0.08 ^a^	0.038
IMP mg/g	2.70 ± 0.13	2.72 ± 0.15	2.60 ± 0.14	0.317

^a,b^ within a row with different superscripts mean significant differences at α = 0.05 level. Values are presented as means ± standard deviation (*n* = 6). L*, lightness; a*, redness; b*, yellowness; IMF, intramuscular fat; IMP, inosine monophosphate.

**Table 5:** Effect of different levels of rutin on serum biochemical indexes of Qingyuan partridge chickens.

Items	Rutin, mg/kg			SEM	*p*-Value
	0	200	400		
TP, g/L	38.79	39.66	40.22	1.240	0.906
ALB, g/L	18.91	20.45	20.06	0.453	0.377
CRE, μmol/L	32.19	30.15	31.49	0.754	0.561
BUN, mmol/L	0.60	0.51	0.50	0.028	0.277
UA, mmol/L	112.41	108.79	111.71	5.574	0.966
GLU, mmol/L	12.34 ^a^	10.87 ^b^	10.41 ^b^	0.234	<0.001
TG, mmol/L	7.11 ^a^	4.06 ^b^	4.19 ^b^	0.379	<0.001
TC, mmol/L	2.49 ^a^	2.08 ^b^	1.94 ^b^	0.078	0.004
HDL, mmol/L	0.52 ^b^	0.58 ^b^	0.93 ^a^	0.046	<0.001
LDL, mmol/L	0.49	0.44	0.42	0.013	0.068

^a,b^ within a row with no common superscript mean significant differences at α = 0.05 level. SEM, standard error of mean (*n* = 6); TP, total protein; ALB, albumin; CRE, creatinine; BUN, urea nitrogen; UA, uric acid; GLU, glucose; TG, triglyceride; TC, total cholesterol; HDL, high-density lipoprotein; LDL, low-density lipoprotein.

**Table 6:** Effect of rutin supplementation on FA composition in breast muscle of Qingyuan partridge chickens.

Items	Rutin, mg/kg			SEM	*p*-Value
	0	200	400		
C10:0	0.09 ^b^	0.20 ^a^	0.25^a^	0.020	0.001
C14:0	0.57	0.56	0.55	0.007	0.570
C16:0	24.72	24.22	24.50	0.169	0.509
C17:0	0.15	0.17	0.17	0.005	0.425
C18:0	8.42	8.29	8.62	0.119	0.551
C20:0	0.11	0.15	0.16	0.012	0.215
Σ SFA	34.01	33.54	34.25	0.214	0.412
C14:1 (*n* − 5)	0.11	0.13	0.11	0.007	0.449
C16:1 (*n* − 7)	3.70 ^a^	2.87^b^	2.92 ^b^	0.141	0.014
C18:1 (*n* − 9)	31.63	31.07	32.13	0.215	0.126
C20:1 (*n* − 9)	0.25	0.27	0.28	0.005	0.199
Σ MUFA	35.69 ^a^	34.27^b^	35.41 ^ab^	0.249	0.037
C18:2 (*n* − 6)	23.12	23.52	22.49	0.229	0.186
C18:3 (*n* − 6)	0.19	0.19	0.19	0.006	0.941
C20:2 (*n* − 6)	0.23	0.25	0.27	0.008	0.094
C20:3 (*n* − 6)	0.37	0.37	0.43	0.015	0.138
C20:4 (*n* − 6)	3.87	4.75	4.60	0.193	0.136
Σ n-6 PUFA	27.77	29.06	27.98	0.262	0.092
C18:3 (*n* − 3)	1.27	1.32	1.31	0.026	0.807
C22:6 (*n* − 3)	0.72 ^b^	1.27^a^	0.97 ^ab^	0.084	0.016
Σ *n*-3 PUFA	1.99 ^b^	2.59^a^	2.28 ^ab^	0.085	0.008
Σ PUFA	29.76 ^b^	31.64^a^	30.26 ^ab^	0.312	0.026
Σ UFA	65.45	65.92	65.66	0.317	0.854
Σ PUFA/Σ SFA	0.88 ^b^	0.94^a^	0.88 ^ab^	0.012	0.036
Σ *n* − 6/Σ *n* − 3 PUFA	14.14 ^a^	11.34^b^	12.44 ^ab^	0.430	0.016
Δ9 activity: 16:1 (*n* − 7)/16:0	0.15 ^a^	0.12 ^b^	0.12 ^b^	0.005	0.013
Δ9 activity: 18:1 (*n* − 9)/18:0	3.77	3.77	3.74	0.065	0.976
Δ6 activity: 20:3 (*n* − 6)/18:2 (*n* − 6)	0.016	0.016	0.019	0.001	0.084
Δ5 activity: 20:4 (*n* − 6)/20:3 (*n* − 6)	10.48	13.70	10.76	0.751	0.153
Δ5 + Δ6 activity: 20:4 (*n* − 6)/18:2 (*n* − 6)	0.17	0.20	0.20	0.009	0.194
Δ5 + Δ6 activity: 22:6 (*n* − 3)/18:3 (*n* − 3)	0.57 ^b^	0.98^a^	0.75 ^ab^	0.069	0.040

^a,b^ The values with no common superscript mean statistical significance at α = 0.05 level. SEM, standard error of mean (*n* = 6); SFA, saturated fatty acids; UFA, unsaturated fatty acid; MUFA, monounsaturated fatty acids; PUFA, polyunsaturated fatty acids.

**Table 7:** Effect of rutin supplementation on antioxidant capacity of Qingyuan partridge chickens.

Items	Rutin, mg/kg			SEM	*p*-Value
	0	200	400		
Antioxidation indicators in serum					
MDA (nmol/mL)	7.09 ^a^	4.24 ^b^	5.07 ^b^	0.323	<0.001
CAT (U/mL)	14.17 ^b^	27.15 ^a^	28.30 ^a^	1.632	<0.001
GSH-Px (U/mL)	5644.63	5776.86	5966.94	131.920	0.633
T-SOD (U/mL)	121.81 ^b^	143.79 ^a^	136.09 ^a^	2.981	0.003
T-AOC (μmol/mL)	1.27 ^b^	1.68 ^a^	1.56 ^a^	0.046	<0.001
Antioxidation indicators in breast muscle					
MDA (nmol/mg protein)	2.33 ^a^	1.96 ^b^	1.37 ^c^	0.103	<0.001
CAT (U/mg protein)	11.57 ^b^	13.83 ^a^	12.74 ^ab^	0.353	0.021
GSH-Px (U/mg protein)	70.96	81.92	75.81	2.488	0.204
T-SOD (U/mg protein)	7.24	7.65	7.08	0.209	0.556
T-AOC (μmol/mg protein)	0.10 ^b^	0.16 ^a^	0.14 ^a^	0.007	<0.001

^a,b,c^ The values with no common superscript mean statistical significance at α = 0.05 level. SEM, standard error of mean (*n* = 6); CAT, catalase; GSH-Px, glutathione peroxidase; MDA, malondialdehyde; SOD, superoxide dismutase; T-AOC, total antioxidant capacity.

## Data Availability

The data used and/or analyzed in this study are available from the corresponding authors upon reasonable request.
